# Correlated Conductance Parameters in Leech Heart Motor Neurons Contribute to Motor Pattern Formation

**DOI:** 10.1371/journal.pone.0079267

**Published:** 2013-11-18

**Authors:** Damon G. Lamb, Ronald L. Calabrese

**Affiliations:** Department of Biology, Emory University, Atlanta, Georgia, United States of America; Claremont Colleges, United States of America

## Abstract

Neurons can have widely differing intrinsic membrane properties, in particular the density of specific conductances, but how these contribute to characteristic neuronal activity or pattern formation is not well understood. To explore the relationship between conductances, and in particular how they influence the activity of motor neurons in the well characterized leech heartbeat system, we developed a new multi-compartmental Hodgkin-Huxley style leech heart motor neuron model. To do so, we evolved a population of model instances, which differed in the density of specific conductances, capable of achieving specific output activity targets given an associated input pattern. We then examined the sensitivity of measures of output activity to conductances and how the model instances responded to hyperpolarizing current injections. We found that the strengths of many conductances, including those with differing dynamics, had strong partial correlations and that these relationships appeared to be linked by their influence on heart motor neuron activity. Conductances that had positive correlations opposed one another and had the opposite effects on activity metrics when perturbed whereas conductances that had negative correlations could compensate for one another and had similar effects on activity metrics.

## Introduction

Many critically important behaviors are controlled by neuronal networks called Central Pattern Generators (CPGs) [1]. CPGs underlie many canonical movement patterns which are critical for life, such as respiration [2], locomotion [3]–[7], and circulation, the system on which we focus here. The successful production of these movement patterns requires coordinated muscle activity. Motor neurons driven by these CPG networks have generally been considered followers of the CPG output. More recently, studies have found that motor neurons, in particular leech heart motor neurons, themselves contribute to the production of their output patterns [8]–[10]. While inputs from premotor interneurons of a leech heart CPG are responsible for the majority of the motor neuron output, motor neurons do not simply follow their input: intrinsic properties appear to play an important role [8], although only a few have been specifically studied [9], [10].

There is a growing consensus in the field that neurons have widely different underlying parameters, especially those associated with intrinsic membrane properties, even while maintaining their identity and characteristic activity [11]–[17]. Intrinsic membrane properties, especially those determined by the maximal conductances (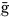) of pools of voltage-gated ion channels, can show in the identifiable neurons of invertebrate nervous systems considerable (up to five fold) animal-to-animal variability, which has been particularly well documented in the crustacean stomatogastric nervous system (STNS) [11], [16]. In the STNS the levels of mRNA that code for these channels have similarly been found to be highly variable [14], [18]. Nevertheless, networks of neurons and even individual neurons can produce tightly regulated activity patterns despite having these widely different underlying parameters. Such animal-to-animal variability in maximal conductances has also been shown for synaptic strengths in the leech heartbeat system [19], [20]. In all these cases, the wide range in values observed could simply be because measured parameters, i.e. the maximal conductance of synapses or ionic currents, do not meaningfully influence the characteristic activity of those neurons, or pairs or sets of these properties could jointly maintain characteristic activity. In particular, the maximal conductances of voltage gated currents that oppose or compensate for one another may be co-regulated or counter-regulated, respectively. In such cases, we should find correlations between these parameters, likely linked by their influence on characteristic activity. Beyond the putative existence of such correlations in the motor neurons we are investigating, it is important to determine how cellular parameters affect the input-output transformation of these neurons. How are leech heart motor neurons coordinated by their inputs, what do they contribute to the patterns they produce, and in particular what role do active membrane conductances play in producing a coordinated motor pattern? Accordingly, we sought a motor neuron model that accurately produced their biological activity and recapitulated the animal-to animal variability of biological neurons.

### Leech Heartbeat System

**Figure 1:**
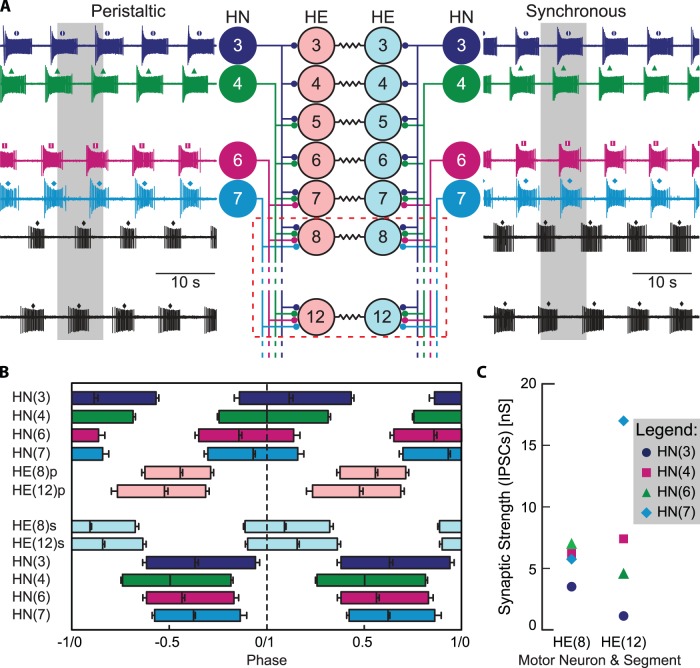
Leech heart motor neuron circuit in segments 3–12 and input/output pattern. A. Simplified circuit diagram for heart (HE) motor neurons depicting the premotor heart (HN) interneurons (ganglia of origin indicated) and the synapses from the former to the latter. Adjacent to each neuron is a representative extracellular recording with the middle spike indicated by a small symbol above each burst. One period is indicated by the grey background bar. Connectivity between interneurons is not shown (for detail, see [20], [57], [58]). Note that ipsilateral midbody motor neurons (e.g., HE(8) and HE(12) above, highlighted with the red broken line) receive the same complement of inputs. B. Relative phasing of first, middle and last spikes in heart motor neurons and interneurons recorded from a single animal, a portion of which is shown in panel A, as reported by Norris [23] and used previously in earlier modeling efforts [9], [10]. Error bars indicate standard deviations. The peristaltic pattern exhibits a strong rear-to-front phase progression in both the interneurons and motor neurons, whereas the synchronous pattern exhibits a minimal phase progression. Note that the HE(8) synchronous (s) and peristaltic (p) motor neuron bursts are nearly perfectly out of phase, unlike the HE(12)p and HE(12)s motor neuron bursts which partially overlap. C. Relative synaptic strength of synapses onto heart motor neurons calculated from spike-triggered averaged IPSCs. Adapted with permission from [23].

We investigated the heart (HE) motor neurons that innervate the tubular hearts of the leech. The leech heartbeat system has been described in great detail previously [19], [21]–[25], so we briefly outline the relevant features of its organization here. The bilateral heart tubes are driven by the ipsilateral member of the pairs of leech heart (HE) motor neurons in ganglia 3 through 18 (HE(3)-HE(18)) of the 21 midbody segmental ganglia [21], [26]. These motor neurons are controlled by barrages of inhibitory synaptic input from a core CPG consisting of 7 pairs of interneurons located in midbody ganglia 1–7 of the animal. The motor neurons in ganglia 8 through 14 receive input from the four ipsilateral premotor interneurons of this core CPG, so the temporal pattern of spikes each receives from each ipsilateral premotor interneuron is identical, except for conduction delays, in particular for the two pairs we specifically focus on, HE(8) and HE(12) (Figure 1A). The heart motor neurons in ganglia 3 through 7 receive input from only a subset of the premotor interneurons, as shown in Figure 1A, as well as input from a pair of unidentified interneurons (not shown), and those in 15 through 18 receive additional input from the rear interneurons [25]. The CPG produces a bilaterally asymmetric pattern, with the premotor interneurons on one side coordinated nearly synchronously while the opposite side is coordinated in a peristaltic rear-to-front progression. These two patterns are imposed on the motor neurons, which produce the corresponding patterns, by the interneurons on each side sculpting the tonic activity of ipsilateral motor neuron into bursts with inhibitory synaptic input. Thus each side of the whole heartbeat system expresses one of two coordination modes of activity at any point in time (Figure 1B): either nearly synchronous activity (referred to as the synchronous mode) which gives rise to near synchronous contractions in the ipsilateral heart tube, or a rear-to-front progression of activity, which gives rise to a corresponding peristalsis in the ipsilateral heart tube (referred to as the peristaltic mode) [27], [28]. The core CPG, and thus the heartbeat system as a whole, alternates between one state (left/peristaltic right/synchronous), where the entire left side of the network is producing the peristaltic pattern and the right side is producing the synchronous pattern, and the reciprocal state (left/synchronous right/peristaltic), with the transitions between these states occurring precipitously every 20–40 beats [29], [30]. Since each motor neuron alternately produces both activity modes, each motor neuron has to produce both input-output transformations, with the pattern produced depending on the temporal pattern of its input. Not only do the switches between modes occur every few minutes, but measurements of synaptic weights show no difference between modes [19], so each motor neuron must produce both patterns without any change in its synaptic weights.

To develop a new model of HE motor neurons, we took advantage of a unique complete input-output data set. Norris et al. [23] recorded simultaneously from all interneurons which synapse onto the midbody heart motor neurons as well as from two motor neurons (HE(8) and HE(12)) themselves during both the peristaltic and synchronous modes, giving us a complete temporal pattern of input and output for 12 animals, one of which was used for this investigation (Figure 1). Furthermore, the strength of these synapses was measured in the same preparation after recording these temporal patterns (see Figure 1C). Measures of the motor neuron activity, in particular phase, duty cycle, and spike frequency, can be used as targets associated with a specific input pattern. The combination of the synaptic strengths with the temporal patterns of spikes from each premotor interneuron gave us a complete input pattern that, when combined with corresponding output targets, allows us to focus on the intrinsic properties of the motor neurons we seek to model. It is important to emphasize here that the only difference between the input to the HE(8) and the HE(12) motor neurons is the synaptic weights of their four inputs in each of the coordination modes, although there is a small offset in spike timing of 80 ms due to conduction delay. The requirement that each model motor neuron must be capable of producing both the peristaltic and the synchronous output patterns appropriate to its segment and the individual animal dataset give us the ability to constrain our models with electrophysiological targets specific to both the peristaltic and synchronous input patterns with which they are simulated.

Previous modeling work has created model leech heart motor neurons which qualitatively captured some of the activity pattern features of those found in the living system, but had difficulty achieving the appropriate phase within a reasonable window [8]–[10]. This heart motor neuron model simplified the morphological complexity into a single isopotential compartment and contained an incomplete complement of membrane conductances. We built upon these modeling efforts and developed a multi-compartmental leech heart motor neuron model which compromised between capturing morphological complexity and reducing computational complexity and included all active conductances believed to be present in the living system. This model was parameterized by the maximal conductance densities of the active membrane conductances and the electrical coupling as described in the Methods. Each instance of the model had a unique set of specific values for each of these maximal conductance densities. Rather than hand tune or attempt a deterministic multi-target optimization algorithm, we used an evolutionary algorithm to find model instances which achieved our target ranges on our fitness metrics. Evolutionary algorithms, including the specific algorithm we used [31], [32], have been shown to be efficient at identifying good model instances, although they are typically stochastic and not guaranteed to be successful [33]–[35]. To generate and evaluate these model instances, we used an input-output dataset from a single animal and the corresponding targets on each of our metrics. Because we had input-output data for both HE(8) and HE(12) heart motor neurons, we were also able to examine possible differences between them to generate predictions of general properties of leech heart motor neurons.

## Methods

**Figure 2:**
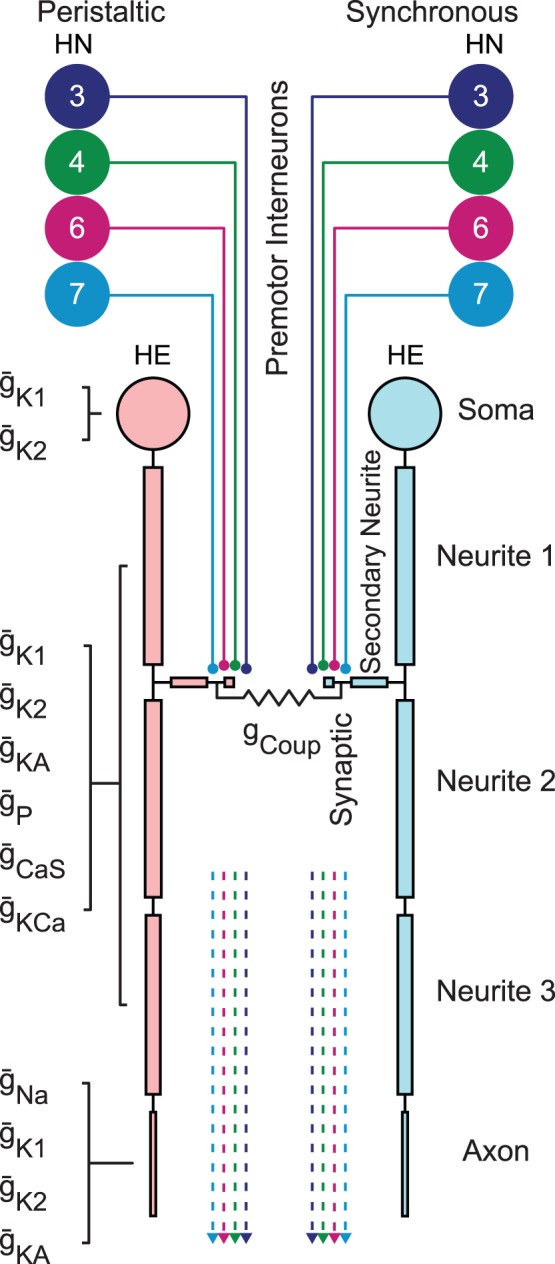
Heart motor neuron model. A pair of electrically coupled 7-compartment model heart motor neurons with their inhibitory inputs shown. The inputs to each model motor neuron were the prerecorded input spike times from the premotor interneurons (delayed based on the motor neuron pair’s ganglion of origin), and the relative strength of the synapses. The soma, neurite and axon compartments contained active conductances in addition to the passive membrane capacitance and leak conductance, whereas the secondary neurite and synaptic compartments were only passive. The soma compartment contained a g_K1_ and g_K2_; the neurite compartments (Neurite 1, Neurite 2, Neurite 3) each contained g_K1_, g_K2_, g_KA_, g_P_, g_CaS_, and g_KCa_; and the axon compartment contained g_Na_, g_K1_, g_K2_, and g_KA_. The synaptic compartment contained synaptic elements and was electrically coupled (g_coup_) to the contralateral heart motor neuron. An instance of the abstract model was defined by specific values for the maximal conductance (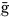) density of each conductance shown above.

We developed a multi-compartmental leech heart motor neuron model parameterized by the maximal conductance (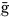) densities of voltage-gated membrane currents and the electrical synapse between the two neurons of each pair (Figure 2). We then used a multi-objective evolutionary algorithm to generate a large number of model instances, each defined by the specific parameter values. Each model instance was simultaneously simulated as four neurons in two pairs with the same parameter values, the heart motor neurons in ganglia 8 and 12 (HE(8) and HE(12)), and the resulting membrane voltage traces were evaluated with the quantitative fitness metrics detailed below. We then examined the distribution of model instances in parameter space, the sensitivity of fitness metrics to parameter perturbation, and the response to injected current of quantitatively good (i.e., within target ranges on our fitness metrics) model instances.

### Simulation and Analysis Framework

**Figure 3:**
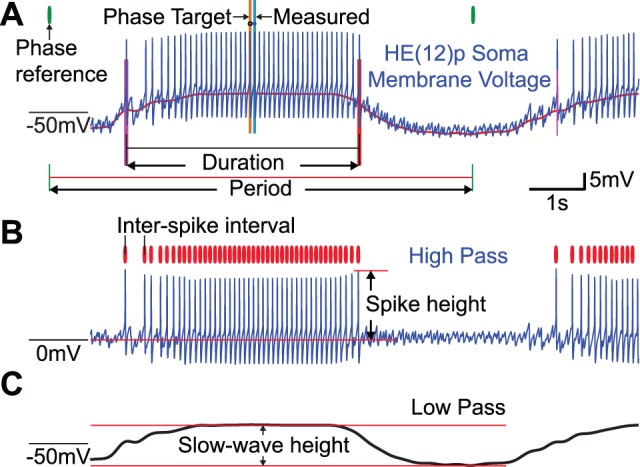
Fitness metrics used to quantitatively evaluate model output. The soma compartment membrane voltage was split into low pass and high pass components, spikes were identified, and 5 fitness metrics were calculated: phase, duty cycle, mean intraburst spike frequency, mean spike height, and slow-wave height. A. The unfiltered soma compartment membrane voltage (blue trace) with the first spike (fuchsia vertical line) and last spike (red vertical line), phase reference (green vertical lines), phase target (black circle and ochre line), and measured phase (blue dot and topaz line), burst duration (black horizontal line), and period (red horizontal line) indicated. The low-pass trace shown in panel C is in red behind the primary trace. The relative phase of the middle spike was calculated with reference to the middle spike of the reference HN(4)p interneuron as shown in Figure 1. The duty cycle was calculated from the phase of the last spike minus the phase of the first spike. All phases were measured with reference to the HN(4)p middle spike. B. High-pass filtered soma compartment membrane voltage (blue trace) with identified spikes (red vertical lines) and spike height indicated. Spike frequency is the inverse of the inter-spike interval within the identified burst. The spike height was defined as the value of the high pass trace at the peak of each spike within the burst. Both the spike height and spike frequency were averaged within each burst. C. Low-pass filtered soma compartment membrane voltage (black trace) with slow-wave height defined as filtered voltage difference at the middle spike relative to the minimum voltage in the inter-burst region.

All model instances were implemented in the general neural simulation system (GENESIS) version 2.3 [36], and were simulated with a time step of 0.05 ms using the Crank-Nicolson [37] method of the Hines solver for all objects except the electrical coupling, which was solved with the exponential Euler solver. The resulting soma compartment membrane voltage was recorded with a time step of 0.5 ms and then analyzed with a suite of custom MATLAB® functions to detect bursts and compute the fitness of each model instance (see Figure 3). The fitness values for all model instances in each generation were passed to a multi-objective evolutionary algorithm [31], [32], [38], which was implemented in C++ and unchanged except for being adapted to interact with our simulations. These three components were coordinated with Bash shell scripts. Although our framework could run on a desktop computer, we took advantage of its inherently parallel structure, which ensured that each model instance could be simulated and analyzed entirely in isolation, and conducted our evolutions on a high performance computing cluster (Ellipse, Emory IT, ∼1024 nodes). The model and associated input/output files will be uploaded to ModelDB upon publication.

### Heart Motor Neuron Model

The heart motor neuron model constructed here using GENESIS 2.3 [36] consists of 7 isopotential compartments whose physical dimensions approximate the surface area found in adult leech heart motor neurons as estimated from confocal reconstructions and previously published morphology [39] as shown in Figure 2. In heart motor neurons, a single main neurite tapers from the soma through the ganglion and out into the periphery as an axon. From the main neurite in the ganglion emerge many secondary neurites that branch extensively and form the input regions of the neuron. We approximated the main neurite and axon using four distinct cylindrical compartments with diminishing diameters whose lengths were set to a maximum of 1/10 the passive electrotonic length constant. The most distal compartment represented the spike initiation zone and axon and is here called the axon compartment. Although there exist no experimental data specifying the exact spike initiation zone, it must be sufficiently distant so that spikes are relatively small when recorded in the soma, and we adjusted the total length of the neurite and axon compartments to achieve an attenuated spike height. The complex structure of the arbor of secondary neurites emerging from the main neurite was approximated by two linked compartments: a passive secondary neurite compartment linked between neurite compartments 1 and 2, and a distal synaptic compartment. Each compartment was linked through an axial resistance determined by its diameter and length to its parent compartment. We thus had a spherical soma compartment (diameter  = 40 µm), three neurite compartments (neurite 1 diameter  = 10 µm, length  = 115 µm; neurite 2 diameter  = 9 µm, length  = 110 µm; neurite 3 diameter  = 8 µm, length  = 100 µm), an axon compartment (diameter = 3 µm, length = 58 µm), a secondary neurite compartment (diameter  = 5 µm, length  = 20 µm), and a synaptic compartment (diameter  = 5 µm, length  = 5 µm), see Table 1. The passive parameters were set to a specific membrane resistance of 1.1Ωm^2^, a leak reversal potential of -40 mV, a specific axial resistance of 0.25Ω/m, and a specific capacitance of 0.02 F/m^2^, resulting in input resistances measured in the soma of ∼70 MΩ, which is within the input resistance range observed in the living system [40].

**Table 1:** Dimensions and upper bounds of conductance densities allowed in the MOEA.

Compartment	Length(µm)	Diameter(µm)	Na(S/m^2^)	P(S/m^2^)	CaS(S/m^2^)	K1(S/m^2^)	K2(S/m^2^)	KA(S/m^2^)	KCa(S/m^2^)	Coup(nS)
Soma	40	40				25	25			
Neurite 1	115	10		9.5	0.5	375	375	50	50	
Neurite 2	110	9		9.5	0.5	375	375	50	50	
Neurite 3	100	8		9.5	0.5	375	375	50	50	
Secondary Neurite	20	5								
Synaptic	5	5								10
Axon	58	3	3500			500	500	750		

Conductance densities are in Siemens per square meter (S/m^2^) except for Coup, which is the upper bound of the static conductance for the electrical coupling in nS.

We then distributed both established conductances and a new calcium-sensitive potassium conductance according to our best estimate of their distribution while still minimizing the number of model parameters by only placing conductances where they were believed to be located and by constraining the neurite compartments to have the same conductance density [8], [34], [40]. Each isopotential compartment was modeled in the Hodgkin-Huxley formalism with:
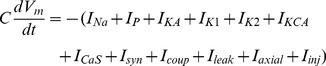
(1)


Where all active conductances were modeled as Hodgkin-Huxley style membrane conductances with the general formula:
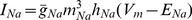
(2)


The specific formula for each membrane conductance is given in Table 2. Both m and h follow the form:
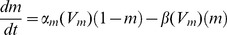
(3)


**Table 2:** Hodgkin-Huxley style membrane conductance formulae.

Conductance	Formula
Leak	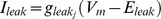
Na	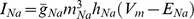
P	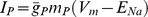
CaS	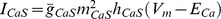
K1	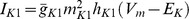
K2	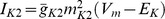
KA	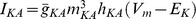
KCa	

**Figure 4:**
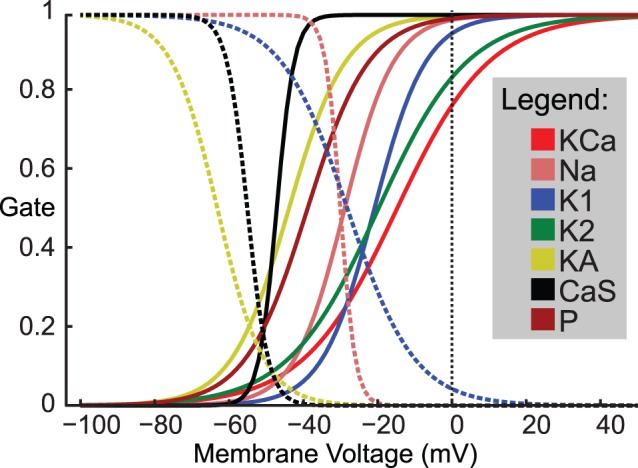
Steady state activation and inactivation curves for voltage-gated conductances. The activation and inactivation gating variables for each of the 7 active membrane conductances used are shown. Solid lines represent activation curves and the dotted lines represent inactivation curves. All conductances except the calcium sensitive potassium conductance have been used in previous heartbeat system models [8], [57].

Genesis 2.3 uses pre-calculated tables for α and β during the simulation, where both α and β were calculated from steady state (see Figure 4) and time constant curves by:
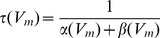
(4)


And
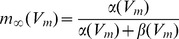
(5)Where those curves (e.g., Figure 4) were specified for each conductance by curves given by:



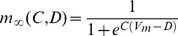
(6)And
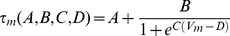
(7)


Except for τ_hNa_, for which h is given by:
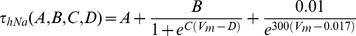
(8)Where A is the baseline value, B scale factor, C is the slope and D is the midpoint of the hyperbolic tangent curve. See Table 3 for the specific values of A, B, C and D for each conductance.

**Table 3:** Voltage-gated conductance model parameter values.

Name	E_rev_	m_∞_		h_∞_		τ_m_				τ_h_			
		C (1/mV)(slope)	D (mV)(V_1/2_)	C (1/mV)(slope)	D (mV)(V_1/2_)	A (s)(τ_min_)	B (s)(τ_max_)	C (1/mV)(slope)	D (mV)(V_1/2_)	A (s)(τ_min_)	B (s)(τ_max_)	C (1/mV)(slope)	D (mV)(V_1/2_)
Na	45	−150	−29	500	−30	0.0001	0			0.004	0.006	−150	−28
P	45	−120	−39			0.01	0.2	400	−57				
CaS	135	−420	−47.2	360	−55	0.005	0.134	−400	−48.7	0.2	8	−250	−43
K1	70	−143	−21	111	−28	0.001	0.011	150	−16	0.5	0.2	−143	−13
K2	70	−83	−20			0.057	0.043	200	−35				
KA	70	−130	−44	160	−63	0.005	0.011	200	−30	0.026	0.0085	−300	−55
KCa	70	−80	−15			0.2	0						

In addition to the established leech conductance models for g_K1_, g_K2_, g_KA_, g_CaS_, and g_Na_ we incorporated, we added a calcium sensitive potassium conductance model based on the g_K2_ conductance with a right-shifted half activation, slower dynamics, and a saturating linear calcium gate (see Tables 2 and 3) to approximate previously published electrophysiological data [40], [41]. To activate the calcium gate of this channel, we added a calcium conductance feeding a calcium pool with a simple exponential decay to baseline (τ = 1.5s) linked to the gate. Earlier heart motor neuron models did not include g_KCa_ or g_CaS_, even though g_KCa_ and g_CaS_ were known to be present [40]. We included g_KCa_ and g_CaS_ not only to better replicate what is found in the living system, but because preliminary modeling suggested that they were capable of altering burst characteristics.

Electrophysiological experiments have provided evidence for the rough distribution of established currents with respect to the compartments used to model these motor neurons. The spikes are small when recorded in the soma, indicating that they are initiated in some distal compartment and are not regenerated or sustained by fast sodium conductances in compartments close to the soma, and thus the axon compartment is the only one which contains a fast sodium conductance, g_Na_. The axon compartment also contains potassium conductances that underlie spike generation and pacing, g_K1_, g_K2_, and g_KA_. In the model, the neurite compartments act as an integrating region, combining the inhibitory input from the premotor interneurons and its membrane properties to suppress or drive spiking in the adjacent axon compartment. The 3 neurite compartments each contained all active conductances except g_Na_: the purely voltage gated potassium conductances g_K1_, g_K2_, and g_KA_, a calcium and voltage gated potassium conductance, g_KCa_, a slowly inactivating calcium conductance, g_CaS_, and a persistent sodium conductance, g_P_. Since these motor neurons were known to express high levels of outward currents as measured from the soma [40], the model soma compartment contains the two potassium conductances likely to be active during normal activity, g_K1_ and g_K2_. These conductances represent the product of the maximal conductance density of each and the surface area of the compartment in which they were contained. The synaptic compartment and the secondary neurite which connects it to the first neurite compartment were both modeled as passive compartments, but the synaptic compartment contained 4 spike-mediated synapse modules, described in detail below, one for each ipsilateral premotor heart interneuron. The synaptic compartment also contained an electrical junction connecting to the contralateral heart motor neuron, g_Coup_. The electrical junction current flow was filtered by a simple rc filter with a time constant of 0.02s applied to the synaptic compartment voltage of each HE neuron of the pair:
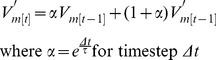
(9)


To keep the number of parameters allowed to vary in the evolutionary algorithm to a minimum, the three neurite compartments had the same conductance densities. Each instance of this heart motor neuron model was thus defined by 13 specific maximal conductance density values: the soma 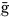
_K1_, soma 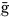
_K2_, neurite 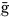
_K1_, neurite 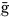
_K2_, neurite 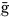
_KA_, neurite 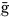
_P_, neurite 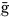
_CaS_, neurite 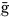
_KCa_, g_Coup_, axon 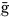
_Na_, axon 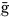
_K1_, axon 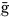
_K2_, and axon 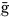
_KA_ (see table 1).

An instance of the heart motor neuron model was hand tuned as a passive model to achieve experimentally determined membrane properties, specifically the time constant and input resistance as determined by a current step injected into the soma compartment. The active membrane conductances were then distributed to the appropriate compartments and their maximal conductances tuned so as to achieve a qualitatively good soma membrane voltage waveform comprising a duty cycle, spike frequency, spike height and slow wave height in the biological range. The initial hand tuned model generated above did not achieve the target phase within the same tight constraints used in the evolutionary algorithm, but did achieve the target range for the other fitness metrics. The evolutionary algorithm was allowed to explore parameter values from 0.1 to 4.9 times those hand tuned values (step size of 0.1) with the exception of g_Coup_, which was limited to less than 3 times the baseline value and used a step size of 0.06, because the initial model value was already at the upper end of what had been observed experimentally, see Table 1 for the maximum allowed value.

Members of each pair of HE neurons, one pair per ganglion, were electrically coupled via g_Coup_. The input pattern contained a temporal pattern (i.e., the spike times for all 8 premotor interneurons, 4 in each coordination mode) and a synaptic weight profile for each pair of heart motor neurons, and was associated with corresponding target values on the fitness metrics described below.

### Input/output Dataset

We chose an input/output dataset from input/output datasets extensively described in previous reports [10], [22], [23]. Briefly, these datasets consisted of simultaneous extracellular (loose cell-attached patch) recordings from all ipsilateral premotor heart interneurons (HN(3), HN(4), HN(6), and HN(7)) in addition to HE(8) and HE(12) motor neurons, a portion of which is shown in Figure 1. These extracellular recordings were long enough to include both the peristaltic and synchronous modes. The interneuron to motor neuron synaptic weights were subsequently measured by dSEVC (discontinuous single electrode voltage clamp) of the heart motor neurons while still recording from the 4 ipsilateral interneurons. From 12 individual animals thus analyzed, we chose 1 input/output dataset with which to develop and examine this model. The two coordination modes were aligned to produce a complete bilateral input spike time pattern with experimentally observed phasing between the two sides (0.51) [22] and were constructed by aligning the 12 synchronous and 12 peristaltic bursts. When used in our simulations, this input pattern was preceded by a 15s silent period to allow model parameters to settle and to ensure that models were tonically active in the absence of synaptic input, extending the total simulation time to 105s. From each motor neuron we calculated fitness metrics by analyzing the 10 bursts which were both preceded and followed by synaptic input for both neurons in each pair. The output targets for phase, duty cycle and spike frequency were those metrics measured in the recorded output pattern and then averaged across the bursts. Slow-wave height and spike height require unclamped intracellular recordings which were not available for every motor neuron, so these targets were taken from established average values from leech heart motor neurons recorded in other experiments. In aggregate, the input/output dataset consisted of spike times and synaptic weight profiles for the 4 premotor interneurons in each coordination mode and fitness targets for the spike height (15 mV), slow-wave height (10 mV), spike frequency (7.37 Hz), peristaltic phase (HE(8): 0.56, HE(12): 0.48), peristaltic duty cycle (HE(8): 0.34, HE(12): 0.46), synchronous phase (HE(8): 0.08, HE(12): 0.11), and synchronous duty cycle (HE(8): 0.44, HE(12): 0.46.

### Inhibitory Input Synapse Model

The inhibitory input synapse model was based on a previously described spike-mediated synapse model [8], [10] with modifications to more closely match the observed conductance waveform of synaptic events in heart motor neurons. This synapse model consisted of two spike-triggered double exponentials, one with a shorter fall time (τ_fall_ = 12.5 ms, τ_rise_ = 4 ms) and the other with a longer fall time (τ_fall_ = 150 ms, τ_rise_ = 4 ms), with the slower component’s maximum conductance set to 0.33 times the faster component (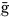
_slow_  = 0.33 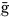
_fast_), resulting in a combined waveform which approximated the shape of inhibitory post-synaptic currents (IPSCs) observed in the living system, which has both spike mediated and graded components [19]. Thus for each spike we have the normalized spike-triggered waveform given by:
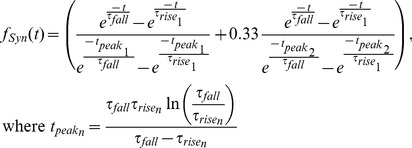
(10)


The synaptic reversal potential was set to -62.5 mV as in prior models [8]–[10] and as measured in the living system [42].The maximal conductances were initially the unscaled relative strengths reported in [19] but were adjusted with scaling factor (σ) to produce a combined inhibitory input that more closely matched that observed in the living system and was capable of sculpting heart motor neuron bursts in our hand-tuned model as in previous models [8]. Furthermore, in the living system the inhibitory synapses from HN onto HE neurons exhibit intraburst synaptic plasticity: IPSCs are initially quite small and then increase to a plateau level before declining towards the end of each presynaptic burst [19]. This plasticity is believed to be due to presynaptic Ca^2+^ accumulation as spike-mediated synapses in the leech heartbeat system are known to be modulated by presynaptic Ca^2+^ entry through LVA Ca channels driven by the slow-wave of presynaptic membrane voltage in heart interneurons [41]. Both presynaptic membrane voltage and free Ca^2+^levels are experimentally inaccessible at present, so we approximated modulation by presynaptic background calcium with a pre-calculated modulation waveform that approximated this rising and falling behavior with an exponential rise from a factor of 0.01 to 1 for 90% of the burst duration and then an exponential decay for the final 10%, similar to previous heart motor neuron models [8]–[10]. For each presynaptic burst (l) starting at time t_0_ we have the waveform given by:
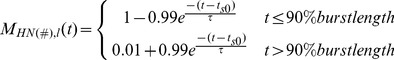
(11)


For each prerecorded input (each HN spike train), we calculated the appropriate rise- and fall-time constants from the average time for the first and final 5 spikes of each burst, for rise- and fall-time constants, respectively, for each premotor interneuron. The synaptic conductance waveforms summate and were weighted by the relative synaptic weight measured in the living system for each HE neuron HN neuron pair, 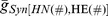, the modulation waveform 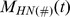, and the scaling factor 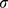 and were combined for each HE motor neuron modeled giving:
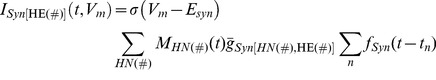
(12)


Thus the complete input pattern delivered to each model instance consisted of a modulation waveform and a train of spikes for each premotor interneuron. These were delayed with a fixed conduction delay of 20 ms per segment (the delay from ganglion 8 to 12 was thus 80 ms).

### Fitness Metrics

Our fitness metrics comprised measures of output attributes that correspond to canonical characteristics of heart motor neuron activity and the overall fictive motor pattern produced. Thus, to evaluate each model instance quantitatively, we used 5 metrics for each neuron being evaluated: phase, duty cycle, intraburst-spike frequency, slow-wave amplitude, and spike height (see Figure 3). Each of these metrics was calculated for every burst of each motor neuron simulated and the resulting values for each burst were then averaged across all bursts for each motor neuron. Because the neural activity we were examining was rhythmic and we aimed to describe the relative phase relationships among the various constituent neurons of the system, we had to select a phase reference, an event within each cycle to define as 0 phase. We used the HN(4) interneuron in the peristaltic mode for this phase reference as in previous reports, e.g. [23], [26]. The period of each cycle was defined as the time between the middle spikes, with the middle spike defined as the median-spike time of subsequent bursts of the HN(4) interneuron in the peristaltic mode as in [23]. The phase of each neuron was defined by the timing of the middle spike of its burst relative to the associated middle spike of the HE(4) interneuron in the peristaltic mode divided by the associated period of the same. The duty cycle of each neuron was defined as the time between the first and last spikes of each burst divided by the associated period. Intraburst spike frequency was calculated for all interspike intervals within detected bursts, thus excluding spurious spikes between detected bursts, and was averaged within each burst to produce that burst’s mean spike frequency. The soma compartment membrane voltage was split into high- and low-pass components using a low-pass filter (1001 point zero-phase FIR filter with a -10 dB cutoff at 1.794 Hz) such that the high-pass component was the remainder when the low-pass component was subtracted from the original waveform. The slow-wave height was the value of the low-pass filtered soma compartment membrane voltage at the trough of the inhibited portion of the cycle minus the value at the middle spike of the burst. Finally, the spike height was the value of the high-pass filtered soma compartment membrane voltage at the peak of each spike within a burst and, as with spike frequency, was averaged within each burst. These 20 values, 5 for each of the 4 simulated neurons, were combined to form 11 metrics that represent the performance of the pairs of HE(8) and HE(12) motor neurons by breaking them into two groups: first, the phase and duty cycle for each of the 4 motor neurons, and second, the basic fitness metrics (the mean intraburst spike frequency, slow wave amplitude, and spike height) which did not differ greatly between each of the 4 motor neurons in each simulation and were averaged across the 4 motor neurons. All model instances had to achieve the target range on the basic fitness metrics to be considered functional. The multi-objective evolutionary algorithm selected the best models independently on each of these metrics, so by combining redundant fitness metrics we avoided overemphasis on model instances which only achieved the target value for one of the basic fitness metrics. If a model instance produced an excellent spike frequency, but was otherwise poor, it would be represented in four fitness metrics if they were not collapsed into one. Since we cared most about phase and duty cycle, and unlike the basic fitness metrics these varied greatly between the 4 motor neurons in each simulation, we did not want such overrepresentation. The target and error threshold for each fitness metric are given in table 4.

**Table 4:** Targets, error thresholds and the mean, minimum, maximum, and standard deviation of set C fitness values for each fitness metric.

Fitness metric	Target	MaxError	Mean	Min	Max	Std
HE(8)p phase	0.556	0.03	0.5288	0.526	0.5369	0.002
HE(8)p duty	0.337	0.1	0.3948	0.3602	0.4161	0.0094
HE(8)s phase	0.077	0.03	0.1054	0.0995	0.107	0.0013
HE(8)s duty	0.436	0.1	0.4752	0.4503	0.4932	0.0085
HE(12)p phase	0.475	0.03	0.482	0.4714	0.4961	0.0042
HE(12)p duty	0.452	0.1	0.5341	0.4814	0.552	0.0132
HE(12)s phase	0.143	0.03	0.1162	0.113	0.1227	0.0021
HE(12)s duty	0.462	0.1	0.5421	0.506	0.5586	0.0084
Spikefrequency (Hz)	7.3682	7	9.6344	5.7768	14.365	1.8715
Spikeheight (mV)	15	7.5	19.0846	13.0818	22.4996	2.0668
Slow waveheight (mV)	10	5	11.6903	7.6234	14.8884	1.1506

### Burst Isolation

Our fitness metrics presume the identification of bursts, and this had to be accomplished in an automated fashion so that an unsupervised algorithm, such as the multi-objective evolutionary algorithm, could be used. We defined bursts as a group of 5 or more sequential spikes between which the interspike interval (ISI) was always less than the minimum interburst interval (IBI). For this investigation, the burst detection algorithm initially set the minimum IBI to 1s. Unfortunately, many model instances did not have clearly defined bursts–instead of a clear separation between bursts evident in the cessation of spiking activity for more than 1s, these model instances merely exhibited a reduction in spike frequency. Although these model instances were almost uniformly deficient on many metrics, we still had to calculate their fitness where possible. In order to calculate the fitness metrics, however, bursts first had to be identified. To do so, the minimum IBI was reduced by a factor of 0.25 until the number of bursts detected matched the number expected for the corresponding input pattern or, failing that, the minimum IBI fell below 50 ms (i.e. below the minimum ISI typically found during normal activity in the living system). When bursts could not be isolated, or where spikes were not detected, the model instance was considered to be bad or failed. Model instances which produced bursts that could be isolated were considered to be at least quasi-functional (our fitness metrics could at least be calculated), even if most model instances did not produce output within the target ranges.

### Multi-objective Evolutionary Algorithm (MOEA)

Model instances were generated and selected by a multi-objective evolutionary algorithm (MOEA) previously used to produce crustacean stomatogastric neuron model instances [31], [32], [38]. Briefly, the first generation was randomized and subsequent generations were bred from exemplars selected independently on each individual fitness metric, with a small amount of random mutation. For example, a model that had an excellent HE(8) peristaltic mode phase (i.e., the best of the present and past generations), but which was unsatisfactory on all other fitness metrics, contributed to the subsequent generation. Since we did not need to create a weighting between the fitness metrics due to the structure of the MOEA, we obviated the complexity and bias that this may produce at the cost of possibly carrying along some poor model instances. The influence of this potential problem was generally negligible because models which were amongst the best on at least one metric were, by definition, satisfactory in some way. Due to the sparse sampling of parameter space (approximately 1e6 models simulated out of the roughly 7e21 model instances that would be required for a brute force approach with the same granularity) and dependence on the random seed for the initial generation, breeding, and mutations, we initiated and combined model instances from a series of 5 evolutions. Model instances which were previously simulated, either in a previous generation or evolution, were not resimulated or reanalyzed, so as to optimize usage of computational resources, but were still treated as if they were by the evolutionary algorithm by reading the previously calculated fitness values.

### Partial Correlations

We evaluated the linear correlational relationships between parameters by examining the partial correlations (ρ) between each pair of parameters [43]. Examining the partial correlation allowed us to evaluate the relationship between parameters while controlling with a general linear model (LM) for the remaining parameters. I.e., for parameters X and Y and remaining parameters Z, we calculated the Pearson’s r between residuals X’ and Y’, that is r(X’, Y’), where X’ = X-LM(X,Z) and Y’ = Y-LM(Y,Z), alternatively expressed as r((X, Y)|Z), the correlation between X and Y given Z. We set the p threshold conservatively with a Bonferroni correction for multiple comparisons to 3.2e-4 and dropped parameters which had at least one p value above this threshold. We then recalculated ρ for the remaining parameters that had all p values below the threshold, leaving us with neurite 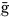
_K1_, 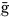
_K2_, 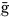
_KA_, 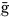
_P_, 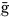
_CaS_ and axonal 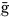
_KA_, 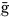
_K2_, and 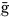
_Na_. The four parameters which were dropped from this comparison, soma 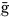
_K1_, soma 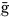
_K2_, g_Coup_ and neurite 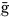
_KCa_, only had one moderate or stronger partial correlation (where |ρ|>0.5), that between 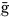
_CaS_ and 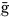
_KCa._ Dropping these four parameters did not substantively influence the recalculation of ρ for the remaining parameters.

### Parameter Variation

We initially defined three sets of model instances: set A, which met all HE(12) and basic metrics targets, set B, which met all HE(8) and basic metrics targets, and set C, which met all our fitness targets. This latter set C thus contains the 431 model instances which were successful as both HE(8) and HE(12) motor neurons. Since there were too many model instances in sets A and B, approximately 39,000 and 4,500, respectively, to perform parameter variation on all model instances in these groups, we selected a randomly chosen subset of set A and set B with 500 model instances for each. The two subsets were selected to ensure that all sets were mutually exclusive, so subset A contained only models which failed on at least one HE(8) metric and subset B contains models which failed on at least one HE(12) metric. Thus, no model instance appeared in more than one subset and 1431 model instances were examined with parameter variation. Each neurite and axon conductance parameter, plus g_Coup_, were systematically varied by ±50% and ±25%, and then evaluated on our fitness metrics.

### Ramp Current Injection

We injected a 5s triangular ramp of current into the soma compartment of the same subsets used for parameter variation (subset A, subset B, set C). After a 10s baseline with no current injected, a 2.5s long ramp from 0 nA to -0.5 nA and then a 2.5s long ramp from -0.5 nA to 0 nA was injected. This was done in the absence of input from the premotor interneurons but with coupling present. We examined and then calculated the change in spike frequency with respect to injected current as well as the last spike time of the downward portion and first spike time of the upward portion relative to time of maximum injected current. We then normalized the resulting F/I curves to the maximal spike frequency observed to better visualize the difference between the downward and upward portion of the ramp injection and calculated the best fit line for the first and second half of each ramp with robust least squares (bisquare weighting) regression. We then analyzed how the first and last spikes differed between the three subsets with a one-way MANOVA and followed up with Bonferroni post-hoc tests.

## Results

### Can We Produce Key Functional Characteristics of Living Heart Motor Neurons in a Model?

**Figure 5:**
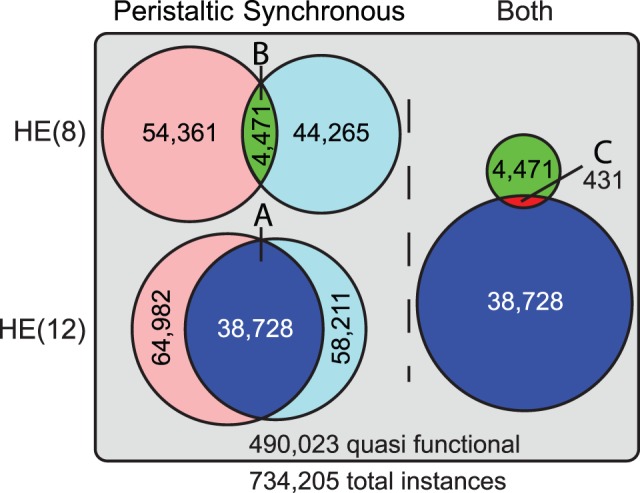
Proportions of model instances, depicted as Venn diagrams, falling within target ranges of fitness metrics. Out of a total of 734,205 model instances which were simulated, 490,023 were at least quasi-functional. The left hand column of Venn diagrams shows the model instances which were capable of producing not only basic fitness metrics (average spike frequency, spike height, slow-wave height) but also the proper phase for the peristaltic or synchronous mode in the indicated heart motor neuron. For example, there were 54,361 models which produced a phase and duty cycle within an acceptable deviation from the target for the HE(8) motor neuron with the peristaltic input pattern, but only 4,471 of these also produced the correct activity with the synchronous input pattern. 431 instances produced output which was within the target range for all metrics, i.e. they produced the target output with all four input patterns: HE(8)p, HE(8)s, HE(12)p and HE(12)s. Three sets were used in subsequent analyses based on the targets they achieved in addition to the basic metrics: set A (blue), the instances which achieved output within the target range for the HE(12) metrics; set B (green), HE(8) metrics; and set C (red), the intersection of sets A and B.

In this study, we successfully created large sets of model instances which replicated, within reasonable tolerances, important functional characteristics of HE(8) and HE(12) motor neurons in the leech heartbeat system: the phase, duty cycle, spike frequency, soma spike height and soma slow-wave height (see Figures 3 and 5). Out of the total of about 700,000 model instances which were simulated across the multiple evolutions, only about 66% produced bursts that could be analyzed, so the range of parameter values we covered was large enough to include regions of parameter space which do not support functional model instances. There were many model instances that achieved all metrics for a given motor neuron (HE(8) or HE(12)) but for only one of the two input modes (peristaltic or synchronous) but not both. These instances cannot be considered functional because the living motor neurons produced both output patterns used as targets [24]. Of the ∼500,000 quasi-functional instances that could be analyzed, 8% were functional HE(12) model instances (set A), just under 1% were functional HE(8) models (set B), and about 0.06% were within the target range for all metrics (set C). Approximately 10% of good HE(8) model instances were able to achieve all target ranges, whereas only 1% of good HE(12) model instances did so. These observations suggest that the ability of the model to produce the desired HE(8) activity put more stringent constraints on parameters than did the HE(12) activity, but that if a model instance could achieve the desired HE(8) activity then it was also likely to achieve the desired HE(12) activity.

The model instances in set C were by definition quantitatively able to replicate the pattern observed in the living system. Furthermore, the soma membrane voltage waveform qualitatively resembled that recorded from HE motor neurons in the living system. We thus produced a varied set of model instances that appear to be a good representation of HE motor neurons with which to investigate the distribution of and relationship between maximal conductance parameters.

### What are the Roles of the Conductances?

**Figure 6:**
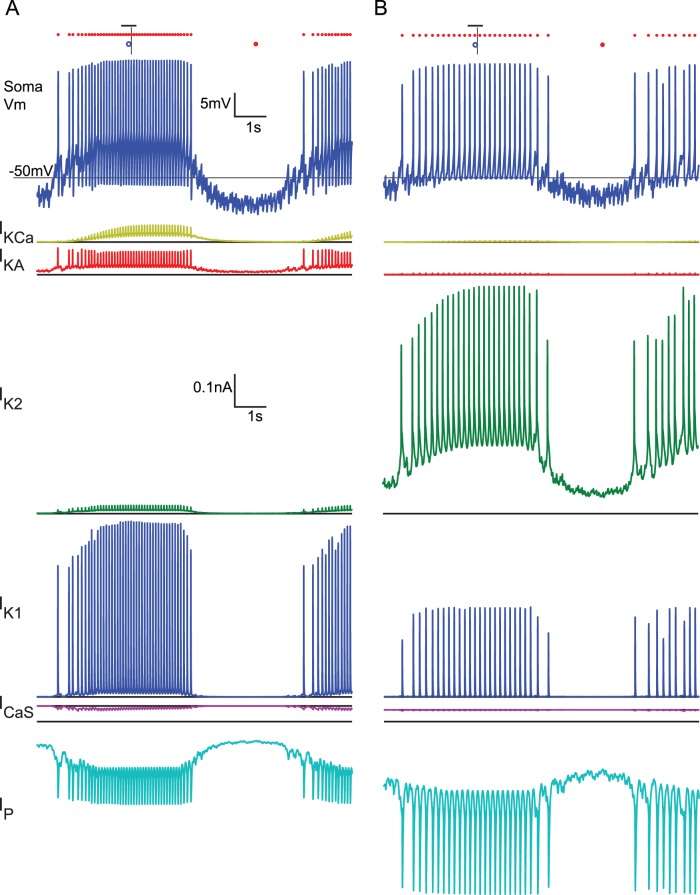
Membrane currents and soma membrane potential in the neurite 2 compartment of two model instances. All actively gated currents (I_K1_, I_K2_, I_KA_, I_P_, I_CaS_, I_KCa_) in the neurite 2 compartment from two representative HE(12)p model instances. Traces are offset for clarity and the solid black line indicates the zero reference for the corresponding trace. On the soma voltage trace, small red circles indicate identified spikes, large red dots indicate the phase reference, blue circles indicates target phase,the short vertical black lines indicate measured phase and the horizontal black line indicates the target range. A. Representative model instance from set C with low 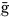
_K2_ and 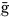
_P_ but 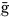
_KA_ in the middle of the allowable range. B. Extreme model instance selected from set C with high 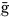
_K2_, near maximum 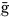
_P_ for set C, and low 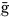
_KA_.

The voltage-gated ionic currents, along with passive leak current, synaptic input, coupling, axial current and capacitive current, interact through their influence and dependence on the membrane voltage in complex ways. Figure 6 shows examples of neurite current flows for two instances of a HE(12) motor neuron from set C, where Figure 6A is a typical model instance and Figure 6B is an extreme case which is dominated by I_P_ and I_K2_ with all other active currents small. The dominant feature of our model is the rhythmic barrages of inhibitory synaptic currents that punctuate the normal tonic activity of the model motor. As we can see from inspection of the individual ionic currents, the currents contributed to the model neuron’s activity pattern as generally expected, but I_KA_ in the neurite was present throughout the burst and during inhibition rather than just between spikes. We examined the currents during three regions of the cycle: during the inhibited portion, during the burst, and during the transitions between inhibition and bursting.

During inhibition, I_CaS_, I_K1_, and I_KCa_ were not activated, but I_p_, I_K2_, and I_KA_ were present. The primary influence these currents had was shifting the baseline membrane voltage according to the size of I_P_ relative to I_K2_ and I_KA_. In the exemplary model instances shown in Figure 6, the slow-wave was smaller in the case shown where I_p_ was predominantly opposed by I_K2_ (Figure 6B), and I_p_ remained more activated during inhibition than I_K2_. In the case where I_p_ was predominantly opposed by I_KA_ (Figure 6A), the slow-wave was larger. The baseline currents, especially the balance between I_K2_ or I_KA_ and I_P_, were critical for these model instances to spike tonically when they were not inhibited and thus to form recognizable bursts. If there was insufficient I_P_, or if it was opposed by too much I_K2_ or I_KA_, then the model instance was not sufficiently excitable to spike tonically without extrinsic current. During bursts the model instances reached a stable tonic level of activity, although there was a small amount of spike frequency adaptation as I_KCa_ and I_K2_ increased throughout the burst. The relative proportions of those baseline currents in the absence of inhibition strongly influenced the spike frequency, the duty cycle, and, to a lesser extent, the phase, as revealed more clearly in the sensitivity analysis below. The spike shape, especially the undershoot, was substantially determined by the faster currents I_KA_ and I_K1_, and model instances with low levels of those currents had less substantial undershoots.

At the beginning and end of inhibitory barrages, we saw the voltage-sensitive dynamics of these currents come into play. At the end of an inhibitory barrage, I_KCa_ was almost totally absent, but I_KA_ and I_K2_ quickly began to activate more fully, as did I_P_. I_P_ quickly rose to its baseline level, as did I_KA_, whereas I_K2_ took slightly longer to reach its baseline level, resulting in an earlier first spike in Figure 6B. The net result of the remaining synaptic inhibition and the further activation of these outward currents was a buildup in spike frequency rather than an immediate jump to the maximal tonic spike frequency. At the onset of an inhibitory barrage, I_KCa_ had reached a steady baseline and contributed to lowering the spike frequency. The outward currents, especially I_K2_, began to deactivate, which could prolong spiking. As we see in Figure 6B, the combination of deactivation and a reduced driving force resulted in less opposition to I_P_ and an additional spike, thus prolonging the burst. The dynamics of the currents as inhibition ends and begins had a primary effect on the first and last few spikes, respectively, and thus also the duty cycle. However, their influence on the first and last few seconds of spikes could also shift the phase of the burst. Although a few spikes at the beginning and end predominantly influenced the duty cycle, the phase tolerance was approximately 2 interspike intervals for the average spike frequency of model instances in set C (1.37 to 3.4 spikes, mean of 2.28), so it did not take many spikes to shift the phase outside the target range. We further explored the difference between the onset and termination of inhibition between model-instance sets with injected hyperpolarizing current ramps below.

### How are the Maximal Conductances Distributed in Parameter Space?

**Figure 7:**
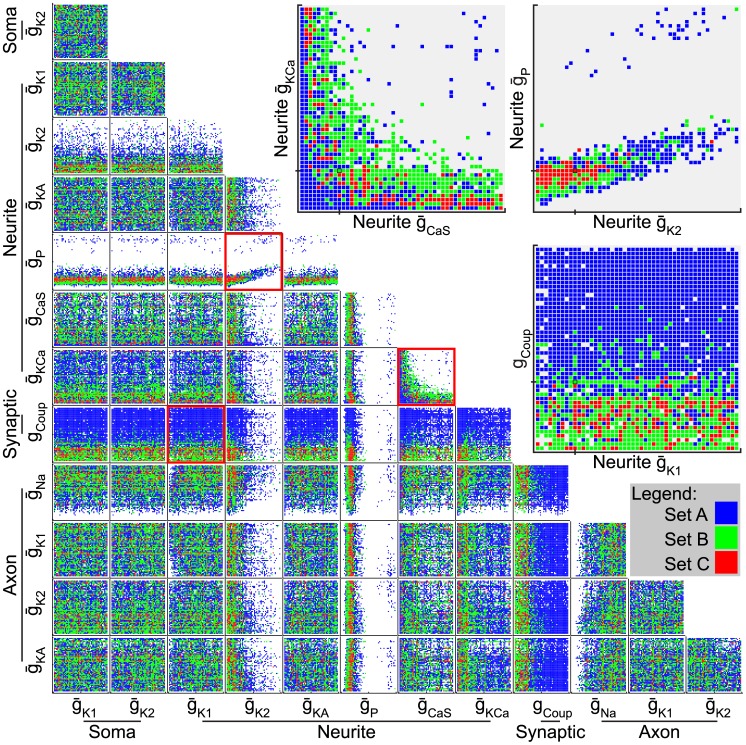
Effect of parameter interaction on fitness set. Functional model instances projected onto 2-D parameter space for each pair of parameters are shown for sets A (blue), B (green), and C (red). Each subplot is a layered scatter plot of set C over set B over set A of the parameter values of functional model instances for each pair of parameters. Each point may represent many models. Axes are from the minimum to maximum allowable value for the parameter indicated. No clear relationship between pairs of parameters was obvious upon inspection in most cases, but some did present interesting structures. The highlighted subplots show three particularly interesting relationships that are apparent upon inspection (shown as insets above right): 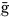
_K2_ appears correlated with 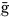
_P_ and is somewhat restricted in its distribution; 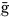
_KCa_ and 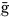
_CaS_ appear to form a non-linear relationship; and electrical coupling (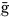
_coup_) is generally restricted to lower values in HE(8) motor neurons (set B) than in HE(12) motor neurons (set A). Black tick marks on the axes and boxes on highlighted subplots indicate the baseline model’s parameter value.

We next turned our attention to how the model instances were distributed in parameter space, or how the parameter values for successful model instances were related to each other given that the maximal conductances showed a large range of values. We found it difficult to directly visualize the potential interactions between parameters when considering the distribution of model instances represented as points in the full 13 dimensional parameter space, so we considered each pair of conductances one at a time. To do so, we examined the 2d projection of the acceptable model instances from sets A, B and C, which contained model instances that achieved the target range on the basic fitness metrics, as well as for HE(12) (Set A), HE(8) (Set B), and both HE(8) & HE(12) (Set C) fitness metrics, respectively (Figure 5). In Figure 7 we show overlaid scatter plots for three sets with set A in blue under set B in green under set C in red for each pair of conductance parameters. Starting first with the gross trends which were apparent upon inspection, we saw that neurite 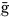
_P_ was strongly limited in range in all three sets of model instances to a small window about the value used in the base model. When hand tuning our base model, we found that model activity was highly sensitive to neurite 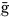
_P_. When neurite 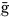
_P_ was too high, the model instance could not be sufficiently inhibited to terminate firing and when it was too low the model instance would rarely spike, let alone form bursts. Next, we found that neurite 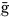
_K2_ tended towards the lower portion of its allowable range, although the restriction was stronger in sets B and C than in set A. As we saw in Figure 6, neurite 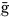
_K2_ could oppose the effect of neurite 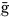
_P_, and the influence of neurite 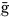
_K2_ on firing was generally opposite that of neurite 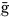
_P_, although our fitness metrics were less sensitive to perturbation of neurite 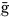
_K2_ than neurite 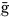
_P_. When 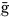
_K2_ was too large, the model instance ceased spiking and when it was small, the model instance’s activity was not sculpted into identifiable bursts.

When we considered the axonal conductances, we found that the range of 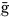
_Na_ was somewhat limited. It could not be too small simply because model instances with very small values of 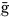
_Na_ could not produce spikes. The remaining axon conductances were not limited, but there was a tendency towards lower values for axon 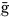
_K2_ that is more obvious in Figures S1–S3. When we examined the electrical coupling in the synaptic compartment, which passed current between the two HE motor neurons in each ganglion, we found an interesting difference between the sets of model instances. In set A, the range of g_Coup_ did not appear to be restricted and there were model instances distributed across the full range of values, whereas in set B, and thus in set C as well, g_Coup_ was restricted to smaller values generally below the hand-tuned value. Larger values of g_Coup_ reduced the phase difference between the coupled neurons as also observed in previous work [8], [10], consistent with the sensitivity results below, and the side-to-side phase difference between the targets for HE(12) motor neurons (0.33) was smaller than that for HE(8) motor neurons (0.48).

Next, we considered the interaction between parameters apparent upon inspection of the plots in Figure 7. First, neurite 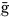
_P_ and 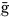
_K2_ appeared to be correlated, which is consistent with what was evident when we inspected the currents in individual model instances in Figure 6. The slow wave components of I_P_ and I_K2_ opposed one another, which mostly mitigated the effects of one another on membrane voltage and excitability. However, even if they perfectly cancelled one another, which they did not, such increases in overall membrane conductance could result in partial shunting, reducing the size of spikes and synaptic inputs as they spread through the affected compartments.

Finally, we come to 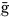
_KCa_ and 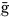
_CaS_, which were only present in the neurite compartments. The distribution of model instances for 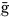
_CaS_ and 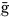
_KCa_ was a non-linear relationship that demonstrates a clear example of why a population approach is well suited to modeling neurons–an average parameter value would likely fall outside of the area of support, an example of a failure of averaging [44].

### How are Currents Correlated?

**Figure 8:**
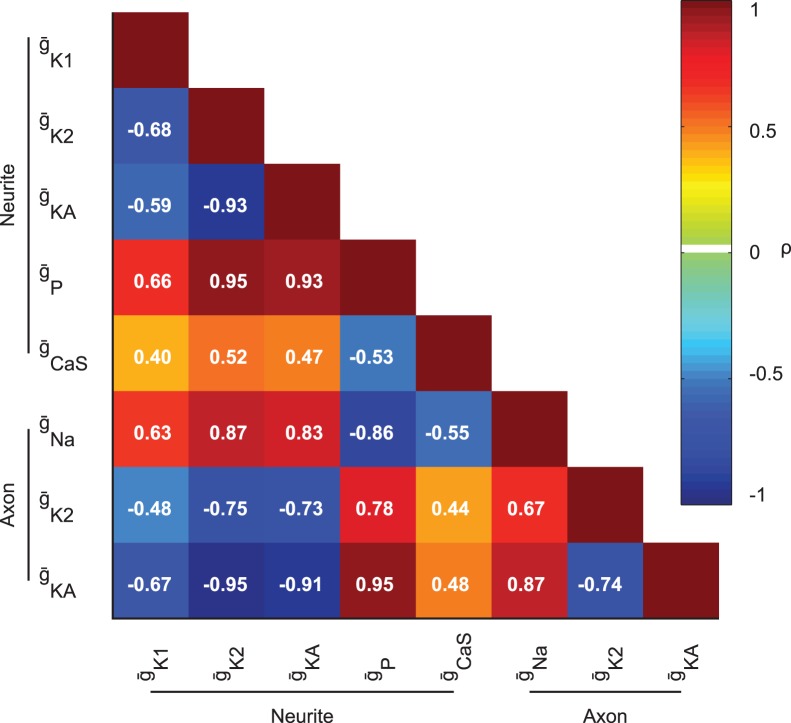
Partial Correlation (ρ) matrix for a subset of parameters. Warmer colors indicate positive and colder colors negative partial correlations. In general, pairs of conductances which oppose each other were positively correlated and pairs that could compensate for each other were negatively correlated. Numbers shown are calculated ρ. All partial correlation shown were significant (p<0.00032).

The partial correlations, the correlations between parameters after compensating for the remaining parameters with a linear model, were then examined for set C. Figure 8 shows these partial correlations for a subset of parameters which were used in the final calculation because they had significant p values (p<0.00032). Neurite 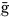
_K2_ was correlated with neurite 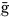
_p_, as expected from our inspection of Figure 7, but neurite 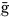
_KA,_ axon 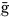
_K2_ and axon 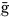
_KA_ were also correlated with neurite 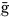
_p_, which was not clear in Figure 7, but the relationship between neurite 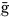
_KA_ and neurite 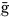
_p_ is illustrated by the currents shown shown in Figure 6. This correlation was somewhat unexpected because, unlike 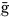
_P_ and 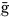
_K2_, 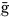
_KA_ inactivates and is generally considered to be responsible for delaying spikes [45], [46]. However, we did observe a baseline window current during bursts, and to a lesser extent during inhibition, in the neurite compartments. The faster potassium current, neurite 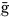
_K1_, was also correlated with neurite 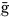
_P_, although this was far weaker and was not present for the axon 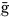
_K1_. Furthermore, there were similar, but weaker, correlations between neurite 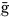
_K1_, 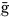
_K2_, and 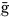
_KA_ with axon 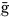
_Na_ and even weaker with neurite 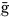
_CaS_. We see a similar pattern with the axon 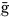
_K2_, and 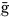
_KA_ correlated with 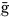
_Na_ as well as with neurite 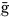
_CaS_, although again the correlations with 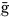
_CaS_ were weak. On the other hand, the outward neurite and axon 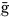
_K2_, 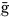
_KA_, and 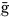
_K1_ conductances were negatively correlated with one another, although these correlations were strongest between 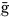
_K2_ and 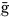
_KA_ for both axon and neurite. We see the same pattern with the inward currents, where 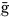
_P_ and 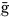
_Na_ and 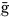
_CaS_ were negatively correlated with one another, although the correlations with 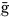
_CaS_ were very weak. Thus the general pattern we observe is that the outward conductance parameters were positively correlated with the inward conductance parameters, whereas the inward conductances were negatively correlated with one another, and similarly the outward conductances were negatively correlated with one another. This pattern of partial correlations held for set A and set B (data not shown), but the partial correlation values were greatly diminished for those sets.

### How did the Parameters Influence the Fitness Metrics?

**Figure 9:**
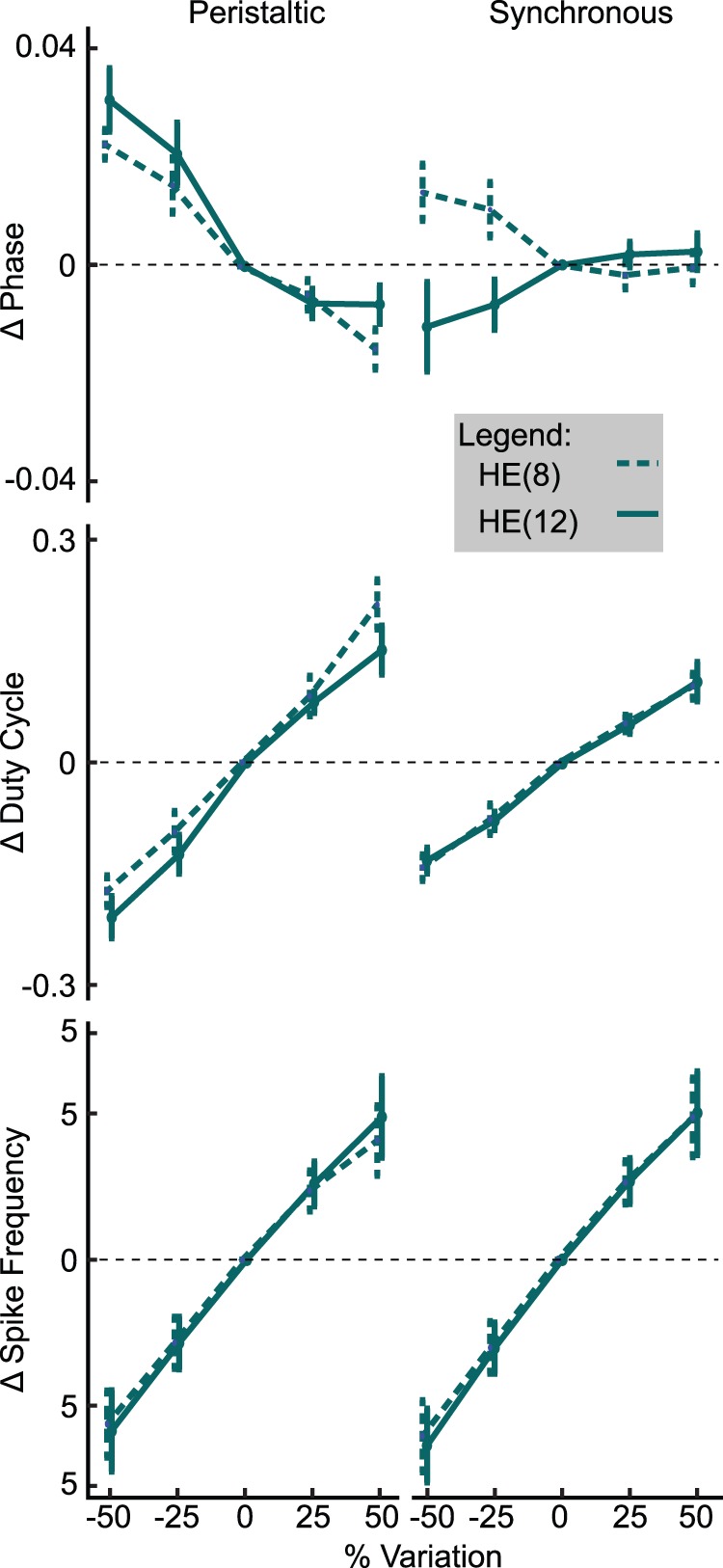
Phase, duty cycle and spike frequency sensitivity to neurite 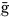
_P_ parameter perturbation. Maximal conductance parameters were perturbed by ±50% and 25% of their initial value for all model instances in set C. The resulting changes in phase, duty cycle and average spike frequency are plotted above for each mode of HE(8) and HE(12) motor neurons. Data shown as mean ± std.

**Figure 10:**
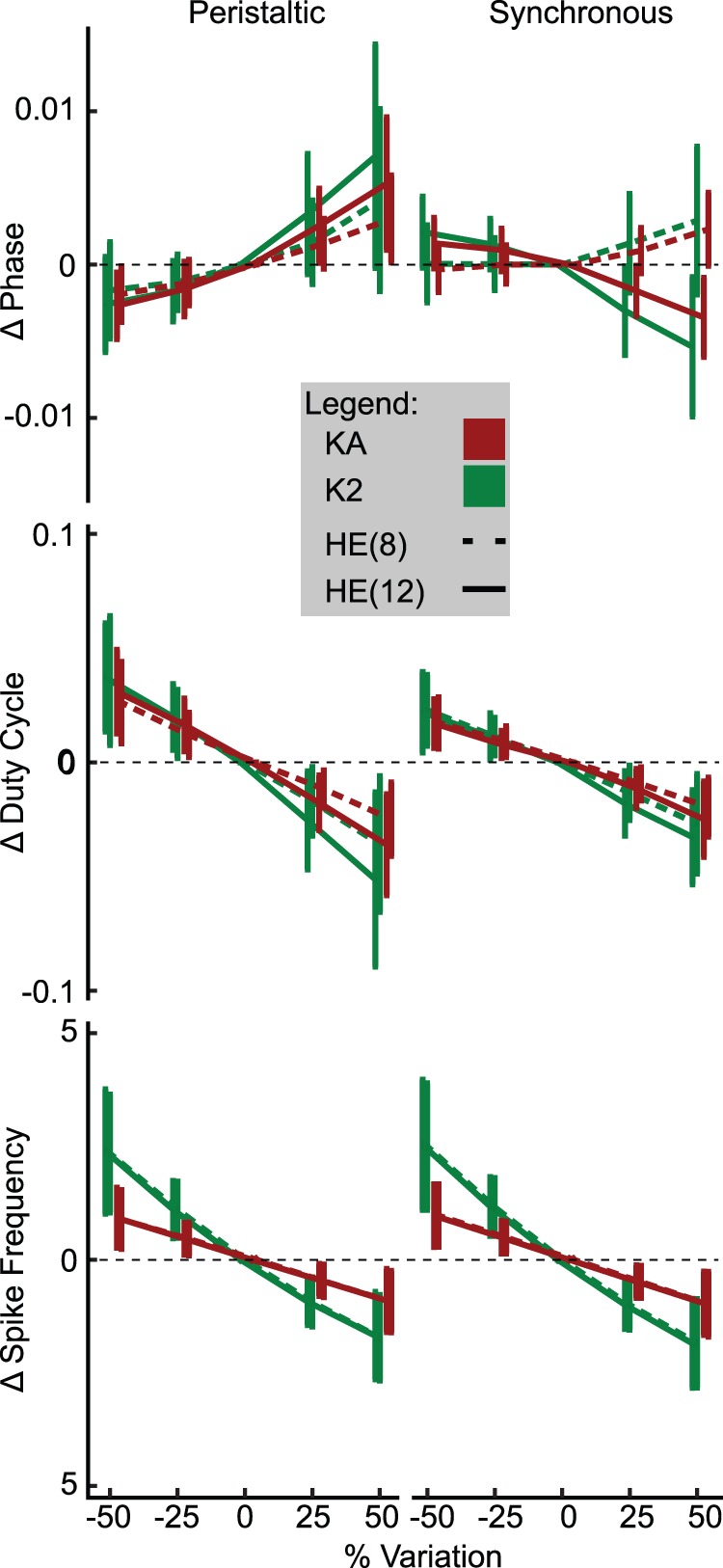
Phase, duty cycle and spike frequency sensitivity to neurite 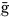
_KA_ and 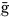
_K2_ parameter perturbation. Maximal conductance parameters were perturbed by ±50% and 25% of their initial value for all model instances in set C. The resulting changes in phase, duty cycle and average spike frequency are plotted above for each mode of HE(8) and HE(12) motor neurons. Data shown as mean ± std.

To explore the influence of parameters on fitness metrics, we perturbed model instances by ±25% and ±50% for each neurite and axon parameter, plus coupling. As it was not feasible to simulate approximately 2 million perturbed model instances in sets A, B and C, we randomly selected 500 model instances each from sets A and B as described in Methods, in addition to all of set C. Only data for set C is shown in Figures 9 and 10, but data were consistent across the subsets. Most of the results were consistent with our general expectation that increases in outward conductances should reduce spike frequency and reduce the duty cycle whereas the opposite should be the case for inward conductances. Manipulation of neurite 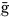
_CaS_, however, followed the pattern of outward conductances because an increase in 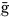
_CaS_ led to increased I_KCa_. Spike frequency was most sensitive to perturbations of 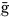
_P_, consistent with our experience when hand tuning the initial model and with the observation that the range of 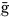
_P_ in good model instances is limited. Duty cycle followed the pattern observed with spike frequency, with increases in outward currents reducing the duty cycle and increases in inward currents increasing it.

We found some unexpected results when we considered the influence of maximal conductance density parameter perturbations on phase. In the peristaltic mode, an increase in neurite 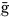
_P_ resulted in a phase delay and a decrease in 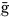
_P_ resulted in a phase advance, and this was consistent for both HE(8)p and HE(12)p motor neurons (Figure 9). In the synchronous mode, however, we observed a phase advance for HE(12) and a phase delay for HE(8) as a result of reducing 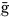
_P_ and a minimal effect of increasing 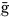
_P_. This result was somewhat confounded by the many model instances which failed when 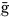
_P_ was reduced by 50% and the extreme excitability induced by increases in 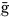
_P_ requiring a reduction of the minimum interburst interval to isolate bursts, thus dropping some spikes. However, we found the corresponding reverse pattern when we examined the effect of perturbing neurite 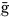
_KA_ or neurite 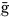
_K2_, (Figure 10). In the peristaltic mode, a decrease in neurite 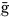
_KA_ or 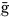
_K2_ resulted in a small phase advance and an increase in these conductances resulted in a larger phase delay. In the synchronous mode, an increase in neurite 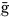
_KA_ or 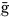
_K2_ resulted in a phase delay in HE(8) and a phase advance in HE(12) motor neurons, consistently in opposition to what was observed for neurite 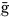
_P_. We also observed a difference between the influence of axon 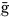
_KA_ and neurite 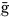
_KA_. I_KA_’s canonical role is to regulate spike frequency, primarily through being active after spikes before inactivating enough to allow another spike to be initiated. In the present model, we saw a result consistent with I_KA_‘s canonical role when we increased the axon 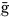
_Ka_, where such an increase resulted in a decreased spike frequency. When we increased the neurite 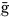
_KA,_ however, we saw a smaller effect on spike frequency, as shown in Figure 10, but a greater influence on phase.

### How Do the Sets Differ in Response to Current Injection?

**Figure 11:**
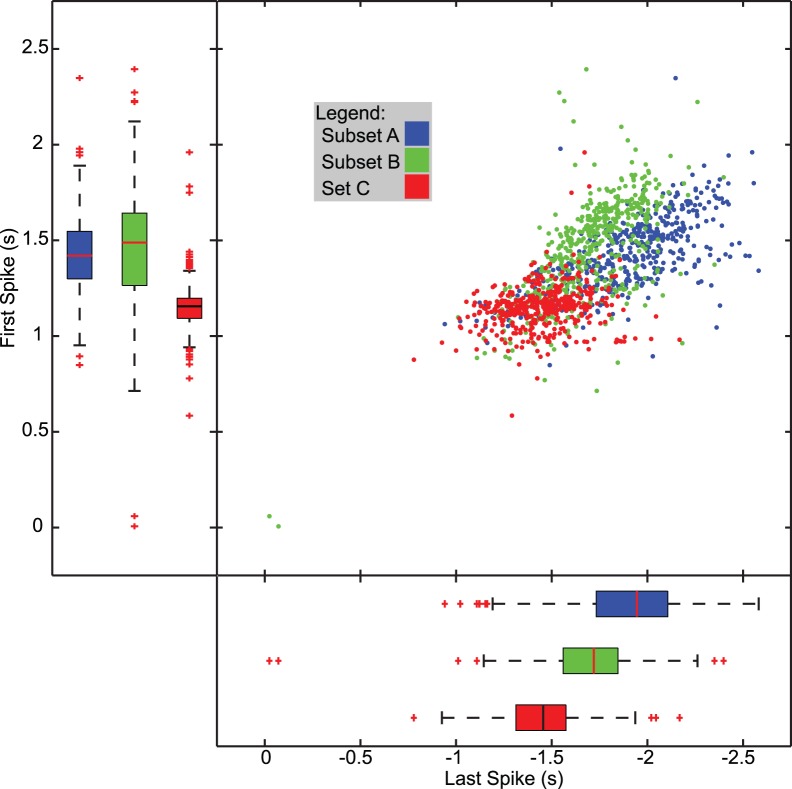
Last and first spike time during F/I ramp injection. First spike vs. last spike relative to the peak hyperpolarizing current injection is shown as scatter and box plots for model instances from subsets A B and set C. Median, 75^th^ and 25^th^ percentile indicated by the center line and edges, respectively, for each box. Red crosses indicate outliers. Model instances were probed with a 5s triangular ramp current from 0 to -0.5 nA and back to 0 injected into the soma compartment. There was a statistically significant difference in last and first spike time for the FI protocol based on set, F(6, 2782)  = 624.171, p<0.0005; Pillai’s trace  = 1.148, partial η^2^ = 0.574. Post hoc tests (Bonferroni) showed a significant difference between set C and subset A (p<0.0005) as well as between set C and subset B (p<0.0005) for the first spike time, but not between subsets A and B (p = 0.111). There was a significant difference between all sets/subsets for the last spike time (p<0.0005).

We then explored how the model instances differed between the sets A, B, and C with respect to their intrinsic properties. To do so, we used the same subsets as were used for parameter perturbations, subsets A, subset B and set C, and examined their activity during injections of hyperpolarizing current ramps that serve as a rough approximation of the inhibitory synaptic input. We measured the spike frequency vs. injected current and the time of the last spike during the down ramp and the first spike during the up ramp of triangular hyperpolarizing current. Even though the model instances produced a wide range of spike frequencies (from 5.78 Hz to 14.37 Hz, mean of 9.63 Hz for set C), we observed that during the down ramp, when the hyperpolarizing current is increasing, the spike frequency was consistently lower than on the up ramp (see figure S4), when the hyperpolarizing current was reducing. This was the case for all three subsets, but set C appeared more tightly constrained than subset A or subset B, having a lower spike frequency on the down ramp (weighted R^2^ and σ from the robust regression were: subset A down (0.639, 0.1367), subset B down (0.775, 0.1262), subset C down (0.865, 0.0884)). We then examined the last and first spikes relative to the trough of hyperpolarizing current to measure their excitability and ability to spike during hyperpolarization and recover from hyperpolarization due to current injection, and determine how the three groups differed; see Figure 11. The model instances from subsets A and B were more widely distributed, consistent with what was observed for the F/I relationships. When examined statistically, we found that there was a significant difference between sets in the last and first spike times (F(6, 2782)  = 624.171, p<0.0005; Pillai’s trace  = 1.148, partial η^2^ = 0.574). The set C model instances continued to fire longer than either subset A or B model instances (Bonferroni, p<0.0005), and subset B continued to fire longer than set A (Bonferroni, p<0.0005). On the up ramp, set C resumed spiking earlier than either set A or set B (Bonferroni, p<0.0005), but subset A and subset B did not differ significantly (p = 0.111). These results suggest that the ability to fire early when inhibition is waning and to continue to fire later when inhibition is building make a model instance adaptable to different input patterns (e.g., HE(8) vs. HE12).

## Discussion

The broader goal of our research was to understand how neuronal networks can generate coordinated motor patterns and thus coordinated movements, and, specifically, how the leech heartbeat central pattern generator coordinates segmentally repeated motor neurons into the fictive heartbeat motor pattern. Furthermore, we sought to understand better how the intrinsic properties of motor neuron contribute to this input-output transformation. The heartbeat CPG rhythmically inhibits the heart motor neurons, and previous research has shown that, although the majority of the motor neuron output pattern is dictated by this input from the CPG, the heart motor neurons are believed to contribute to pattern formation [9], [10]. We thus set out to develop a heart motor neuron model that more fully captured the complexity of the living system than did previous models, and we successfully developed the first model of HE motor neurons that was capable of quantitatively achieving the target ranges on our fitness metrics, including the phasing observed in the living system.

To develop a more realistic heart motor neuron model, we first constructed a baseline hand-tuned multi-compartmental model and then used a multi-objective evolutionary algorithm to generate model instances (variations of this model) that had differing maximal conductance parameters, but all other properties of each model instance remained the same between instances. These model instances were evolved to achieve target ranges on fitness metrics which captured key output measures or electrophysiological characteristics recorded in the living system. In so doing, the algorithm produced model instances which were capable of quantitatively achieving appropriate target phases and soma membrane voltage waveforms that qualitatively resembled those recorded in the living system.

We focused on how the conductance densities contributed to the fitness of model instances because previous modeling studies implicated intrinsic membrane properties in motor neuron phasing [8]–[10]. We found strong partial correlations between many key conductance densities, in particular between neurite 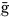
_KA_, 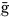
_K2_, 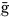
_P_ and axon 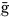
_KA_, 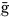
_K2_, 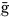
_Na_. Parameters that had strong partial correlations, either positive or negative, appear to be linked by their influence on each of the fitness metrics we used to select good model instances. Conductances that opposed one another had the opposite effects on fitness metrics when perturbed while conductances that could compensate for one another had similar effects.

### Selection of Fitness Metrics

We chose a restricted subset of possible fitness metrics – phase, duty cycle, average spike frequency, spike height, and slow-wave height – with which to evaluate our model and to evolve model instances. Phase and duty cycle are fundamental metrics when describing rhythmically active processes. Furthermore, phase and duty cycle, along with spike frequency, specify the output of these motor neurons–the timing, duration, and intensity of the activity that controls the heart muscle fibers they innervate. The average spike frequency, height, and slow-wave height are important metrics that capture the gestalt of heart motor neurons. In the living system, heart motor neurons are not only identified by their location and soma dimensions, but also by their characteristic activity: appropriately phased bursts of relatively small spikes with moderate spike frequency and a marked reduction in soma membrane voltage during inhibition. Thus, the metrics used in the present study represent the minimal complement necessary to accurately evaluate a heart motor neuron model.

### Parameter Correlation and Regulation

When we examined the resulting model instances we found several interesting relationships between the maximal conductance parameters we allowed to vary in the evolutionary algorithm. Inward and outward currents that opposed one another were generally positively correlated, whereas those which could compensate for the loss of one another were negatively correlated. These relationships held across compartments. These results validate our intuition that conductances which are broadly in opposition, specifically the persistent inward sodium and the outward potassium conductances, could be ratiometrically increased (coregulated) without substantially altering activity. Conversely, outward currents which could partially compensate for one another were counter-regulated while broadly maintaining a characteristic output pattern. In our model, the inward and outward currents had positive partial correlations and thus we would expect them to be co-regulated in the living system provided that the proportions are considered as a larger group than individual pairs of conductances. On the other hand, many of the conductances we found to be correlated have very different dynamics, so it was somewhat surprising to see such strong positive and negative correlations until we considered their influence on the fitness metrics. For example, the target range allowed for phase is small, and achieving it appears to require a coordinated balance between parameters. If we consider a model instance slightly outside of the target range, then shifting into the target range would require coordinated changes to several parameters that individually and in combination shift the phase in the desired direction while not shifting the activity outside of the acceptable range on any other fitness metric. For many models, perturbations of individual parameters helped achieve one target while shifting away from another. Even though the allowable ranges for spike frequency and duty cycle were larger, as were the observed ranges for those metrics in the living system, these metrics are the most sensitive to parameter perturbation, so they could also be driving the observed correlations. The influence on spike frequency in particular appears to be related to the baseline current during the burst, and the conductances with larger baseline currents were more strongly correlated. The prediction that these conductances are correlated and that this is through their influence on the metrics we used to define the model instance sets is supported by a recent investigation in the pyloric CPG of the crustacean stomatogastric ganglion [47]. In that study, a dynamic clamp was used to vary three currents, I_A_, I_h_, and a compound current, I_HTK_, and many of the activity attributes measured were influenced by all three currents investigated, with the influence of combinations reflecting compensatory effects between the currents.

One unexpected aspect of our results is that I_KCa_ appeared to be limited, either through low to moderate values of 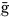
_CaS_ or 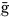
_KCa_. This relationship is likely due to the interaction between 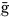
_CaS_ and 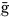
_KCa_ and their influences on the fitness of model instances. The direct effects of 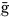
_CaS_ were relatively small compared with 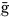
_P_, and 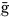
_CaS_ is not limited in range. Even though the direct effects of 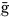
_CaS_ were small, it is of critical importance because the accumulation of Ca^2+^ in each neurite compartment’s calcium pool gates I_KCa_ which, if present at sufficient levels, has an adverse influence on our fitness metrics. I_CaS_ was linked to g_KCa_’s calcium gate through a simple calcium pool model within each neurite compartment. High levels of both 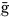
_KCa_ and 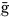
_CaS_ resulted in a high level of I_KCa_, which can prematurely terminate bursts, radically advance phase, reduce the duty cycle, and substantially reduce the spike frequency, all of which result in fitness values outside of the target range. When examined in our sensitivity analysis, increases in 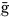
_KCa_ advanced the phase whereas decreases delayed the phase, although the effect was small. The previous single compartmental model’s synchronous phase was delayed relative to the living system [8], so the phase advance resulting from I_KCa_ should have helped achieve the target synchronous phase. However, our results showed that I_KCa_ must be limited. Even so, I_KCa_ may help achieve the F/I results, as the down ramp of the injected hyperpolarizing current resulted in a lower spike frequency than the up ramp when the model instance resumes firing, although the dynamics of all the conductances in the neurite, axon and soma compartments will have to be carefully explored in future research to fully elucidate their relative contributions.

Another parameter which appeared to be tightly restricted is 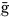
_P_. The distribution of good model instances in parameter space and the extreme sensitivity of the fitness metrics to perturbation of 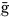
_P_ indicate that this conductance is important. This result is not surprising because neurite 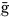
_P_ drives the membrane voltage in the axon compartment, via its direct influence in the neurite compartments, into a range where spikes are initiated in the spike initiation zone in the neighboring axon compartment. The opposition of 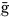
_P_ by 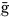
_KA_ and 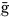
_K2_ appears to be a primary driving factor in the correlational relationships we found, and this relationship is likely due to the baseline currents in the neurite compartments.

In contrast to the previous heart motor neuron model, which predicted that a gradient of electrical coupling that increased towards the rear of the animal would be necessary to achieve the intersegmental phase relationships observed in the living system [8], [9], we found that a coupling gradient is not necessary for proper pattern formation. Coupling conductance values in the range that supports sets B and C would work just as well for set A, so there was no requirement for a gradient. Furthermore, the coupling in sets B and C appeared to be constrained to the range observed in the living system. We did find that the coupling can influence phase by bringing the phases of the two heart motor neurons in each pair closer, but this effect was small. As such, an increasing gradient from the front to the rear of the animal could help achieve the progressively closer phases between the peristaltic and synchronous heart motor neurons, as predicted by prior modeling work, but our results do not indicate that this is required and thus it is unlikely to be a primary feature of heart motor neurons in the living system.

### Neural Identity and the Consequences of Variability

In nervous systems with unambiguously identifiable neurons, such as the leech, in which we can often identify specific neurons by their physical attributes including location, size and morphology, but ultimately by their characteristic activity. We found that attributes of this characteristic activity, as measured by our fitness metrics, are influenced by the conductance densities we allowed to vary between model instances. When we perturbed individual parameters, although some of the fitness metrics might improve (i.e., move closer to their target value) others would move away from the target value. Such a perturbation would typically result in values outside of the target range on at least one metric. The maintenance of multiple attributes of characteristic activity, and thus neural type, requires coordinated changes to multiple conductances that oppose or can compensate for one another, manifesting as partial correlations in our analysis. In systems where the mRNA that codes for the ion channels that underlie membrane conductances has been measured, some correlations have been found to be neural-type specific. For example, in the STNS, the copy number of mRNA coding for hyperpolarization activated non-specific cation current (*IH*, I_h_), a transient potassium current (*shal,* I_A_), two delayed rectifier potassium currents (*Shaw* and *shab*, I_Kd_), and a calcium sensitive potassium current (*BKKCa*, I_KCa_) are correlated with one another in combinations and proportions specific to each neuron type [18]. Furthermore, IH and *shal* are significantly correlated with activity features such as phase mean interspike interval [11], [18], [48] and models of the STNS have shown that linear conductance correlations appear to help maintain such activity features as spike and burst phase, spike frequency and count, and other measures of neuronal type [15], [49], [50]. Even though such correlation can maintain activity features, the cellular cascades responsible are not necessarily activity dependent, as one might initially expect [51], [52].

The input patterns and output targets we used were drawn from characteristic activity patterns under standard conditions. When other input patterns were used in small pilot evolutions, the small number of resulting model instances appeared to follow the same patterns observed in the present data set. The input pattern used here had a middle of the road HE(8)-HE(12) phase progression and was otherwise typical, so we do not believe our results would substantially change given a different input/output data set, but follow-up experiments will have to directly address this question.

In non-standard circumstances – such as the application of drugs, neuromodulation, or perturbation of other cellular parameters – the consequences of intrinsic parameter variability can come to the fore, manifesting as varying responses to perturbation. For example, temperature can affect membrane conductances differently [53]. Simplistically, we can consider the case of two conductances which are strong, but oppose one another, and are exposed to a shift in temperature. If this shift differentially affects these conductances, then the activity pattern it produces can be radically altered or even terminated. For example, if 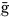
_P_ were to be influenced by modulation or temperature out of proportion to 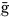
_K2_, then the result would tend toward extremes, either the cessation of spiking activity or spiking through periods of inhibition. Such a differential response to perturbation is exactly what is believed to underlie the results of recent investigations into the effect of temperature on the crustacean stomatogastric ganglion [54], [55]. The differential response of conductances to perturbation is also of concern when we consider the influence of neuromodulators or drugs. Neuromodulators and drugs influence subsets of conductances, but their effects on neural activity is influenced by the extant conductances (ion channels in the cellular membrane) in each neuron. As such, the intrinsic variability of intrinsic properties could lead neurons that appear similar to respond differently to these factors. Recent experimental work in the crab cardiac ganglion has shown disruption of conductances with pharmacological blockers results in differential response between cells, even within a single cell type in an individual animal, and a disruption of normal activity at the network level [56].

### General Conclusion

We developed the first model of heart motor neurons that was capable of producing a quantitatively accurate activity pattern and used it to elucidate relationships between parameters that are necessary to maintain the production of this activity pattern. No model will ever perfectly capture the entirety of what it represents–all models are, in some way, limited. In the case of models of neurons, we accept many reductions and simplifications in order to focus on the particular characteristics we are interested in–for example, we collapse regions of the neuron into isopotential compartments and approximate populations of discrete ion channels with Hodgkin-Huxley style differential equation based models, to name just a few common reductions. Even so, we can produce models with striking predictive power that not only accurately represent specific neurons but also elucidate basic mechanisms controlling activity that apply to them and to neurons in general. In this paper, we have taken advantage of some of the unique characteristics of the leech heartbeat system as well as a population based modeling approach to further elucidate the electrophysiology of motor neurons. The model we have developed provides a large population of model instances with which to perform virtual experimentation, including those involving the manipulation of properties and parameters not experimentally accessible in the living system. Furthermore, the approach we have taken easily generalizes to other neuron types or even small neuronal networks.

## Supporting Information

Figure S1 **Parameter histogram for set A.** Counts are normalized to the total number of model instances in set A. Bin size is 0.04.
(EPS)

Figure S2 **Parameter histograms for set B.** Counts are normalized to the total number of model instances in set B. Bin size is 0.04.
(EPS)

Figure S3 **Parameter histograms for set C.** Counts are normalized to the total number of model instances in set C. Bin size is 0.04.
(EPS)

Figure S4 **Normalized spike frequency vs. injected current.** Model instances in subset A, subset B, and set C were probed with a 5s triangular ramp current from 0 to -0.5 nA and back to 0 injected into the soma compartment. Spike frequency is normalized to maximum spike frequency during ramp protocol. Panels A, B, C contain the data for subset A, subset B and set C, respectively. Black dots and the dashed regression lines represent spikes from the first half (downward portion) of the ramp and colored dots and solid regression lines represent spikes from the second half (upward portion). Panel C compares the regression lines from the three groups. Regression lines are calculated with robust least squares regression (bisquare weighting). Weighted R^2^ and σ for the regression lines were: subset A downward (0.639, 0.1367), subset A upward (0.848, 0.0999), subset B downward (0.775, 0.1262), subset B upward (0.878, 0.0969), subset C downward (0.865, 0.0884), subset C upward (0.837, 0.0999).
(EPS)
